# Circular RNAs as Potential Blood Biomarkers in Amyotrophic Lateral Sclerosis

**DOI:** 10.1007/s12035-019-1627-x

**Published:** 2019-06-07

**Authors:** Ana Dolinar, Blaž Koritnik, Damjan Glavač, Metka Ravnik-Glavač

**Affiliations:** 1 Department of Molecular Genetics, Institute of Pathology, Faculty of Medicine, University of Ljubljana, Korytkova 2, 1000 Ljubljana, Slovenia; 2 Institute of Clinical Neurophysiology, Division of Neurology, University Medical Centre Ljubljana, Zaloška cesta 7, 1000 Ljubljana, Slovenia; 3 Department of Neurology, Faculty of Medicine, University of Ljubljana, Zaloška cesta 2, 1000 Ljubljana, Slovenia; 4 Institute of Biochemistry, Faculty of Medicine, University of Ljubljana, Vrazov trg 2, 1000 Ljubljana, Slovenia

**Keywords:** Amyotrophic lateral sclerosis, Circular RNAs, Differential expression, Biomarkers, Human blood samples

## Abstract

Circular RNAs (circRNAs) are emerging as a novel, yet powerful player in many human diseases. They are involved in several cellular processes and are becoming a noteworthy type of biomarkers. Among other functions, circRNAs can serve as RNA sponges or as scaffolds for RNA-binding proteins. Here, we investigated a microarray expression profile of circRNAs in leukocyte samples from ALS patients and age- and sex-matched healthy controls to identify differentially expressed circRNAs. We selected 10 of them for a qPCR validation of expression on a larger set of samples, identification of their associations with clinical parameters, and evaluation of their diagnostic potential. In total, expression of 7/10 circRNAs was significant in a larger cohort of ALS patients, compared with age- and sex-matched healthy controls. Three of them (hsa_circ_0023919, hsa_circ_0063411, and hsa_circ_0088036) showed the same regulation as in microarray results. These three circRNAs also had AUC > 0.95, and sensitivity and specificity for the optimal threshold point > 90%, showing their potential for using them as diagnostic biomarkers.

## Introduction

Amyotrophic lateral sclerosis (ALS) is a fatal neurodegenerative disease that affects both upper and lower motor neurons, resulting in muscle atrophy, speech difficulties, and respiratory insufficiency [1]. The majority of patients are classified as sporadic (SALS), while 10–15% of patients have known familial disease history (FALS) [2]. Current diagnosis of ALS is based mainly on clinical examination and it can take as much as 1 year to establish a diagnosis after the initial symptoms appeared [3]. Since patients have a mean life expectancy of 30 months [4], establishing a diagnosis represents considerable part of the disease duration. Unfortunately, approximately half of the patients receive an alternative diagnosis beforehand the ALS diagnosis [3]. Thus, reliable biomarkers are an absolute necessity for earlier and more accurate diagnosis of ALS, even more so for the diagnosis of patients with no genetic mutations or familial background. Several fluid-based biomarkers have been already proposed (for recent review on this topic, see [5]). Among them, the most promising are two proteins, neurofilament light chain (NFL) and phosphorylated neurofilament heavy chain (pNFH), that can be detected by immunoassays in cerebrospinal fluid, serum, and plasma [5]. In cases with a mutation in one of ALS-causing genes, diagnosis is confirmed by genetic testing. These mutations are associated with approximately 70% of FALS and 15% of SALS cases [2]. Mutated genes can perturb various biochemical pathways in motor neurons and lead to cell death [6]. The reason for motor neuron death in other cases remains elusive. Several epigenetic mechanisms have been already implicated in the disease development and progression [7]. Among them are also non-coding RNAs with several distinct groups of molecules—micro RNAs (miRNAs), long non-coding RNAs (lncRNAs), and small nucleolar RNAs (snRNAs) [8]. Circular RNAs (circRNAs) represent yet another class of non-coding RNAs that lately gained quite some attention [9–11]. Supposedly arising from back-splicing events during precursor mRNA processing, circRNAs are resistant to RNA exonucleases and thus highly stable in cells [12]. Among versatile functions of circRNAs are also miRNA sponging and RNA-binding protein (RBP) sequestration, both linked to gene regulation [13]. In the process of miRNA-sponging, each circRNA competitively binds multiple miRNAs and reduces their mRNA silencing potential [14]. Similarly, circRNAs can act as RBP-binding sites and scaffolds for protein complexes [14]. Moreover, circRNAs have been already implicated in several neurological and neurodegenerative diseases, such as glioma [15–17], Alzheimer’s disease [18], and Parkinson’s disease [19]. Here, circRNAs acted as miRNA sponges [16, 18, 19] or templates for protein translation [15, 17].

Considering the involvement of miRNAs in the ALS progression and potential role of circRNAs in their regulation, we wanted to determine differential expression of selected circRNAs in patients with SALS and assess their potential use as novel blood-based biomarkers for disease evaluation.

## Materials and Methods

### Samples

Patients were diagnosed with ALS at the Institute of Clinical Neurophysiology, University Medical Centre Ljubljana, Slovenia. Sixty patients (30 females and 30 males) were included in the study, as well as 15 age- and sex-matched healthy controls. Detailed clinical characteristics are shown in Table 1. The study was approved by the National Medical Ethics Committee of Republic of Slovenia and a written informed consent was obtained from all participants.

**Table 1:** Clinical characteristics of patients and healthy controls

Characteristics	Samples		Subset for microarray analysis	
	ALS (*n* = 60)	Healthy controls (*n* = 15)	ALS (*n* = 12)	Healthy controls (*n* = 8)
Sex (M/F)	30/30	9/6	6/6	4/4
Age (years)^a^	67 (35–92)	58 (49–73)	61 (45–70)	53 (53–73)
Age at onset (years)	65 (35–92)	/	59 (44–70)	/
ALS onset (spinal/bulbar/mixed)	45/13/2	/	7/5/0	/
Disease duration (years)^b^	1.5 (0.0–5.5)	/	1.5 (0.5–5.0)	/
Survival time (years)^c^	2.0 (0.5–5.0) *n* = 27	/	2.0 (1.0–5.0) *n* = 9	/
Level of functional impairment^d^	34 (20–48)	/	35 (20–45)	/
Rate of progression^e^	− 1.11 (− 0.03 to – 4.19)	/	− 1.54 (− 0.09 to – 4.19)	/

^a^Age at the time of blood collection

^b^Time from symptom onset to blood collection

^c^Time from symptom onset to death

^d^ALS-FRS-R (ALS functional rating scale revised) points at the time of blood collection

^e^Slope of the linear regression line for ALS-FRS-R points

### RNA Extraction

Peripheral blood mononuclear cells (PBMCs) were isolated from fresh blood using Ficoll density centrifugation (GE Healthcare, Sweden). Collected cells were stored in Qiazol reagent (Qiagen, Germany) at − 80 °C. Total RNA was extracted from collected cells using miRNeasy Mini Kit (Qiagen, Germany) according to the manufacturer’s instructions. The concentration and purity of total RNA were measured with NanoDrop ND-1000 (ThermoFisher, USA).

### Microarray Analysis of circRNA Expression

Microarray analysis of circRNA expression was performed on a subset of 20 samples—12 patients (6 females, 6 males) and 8 age- and sex-matched controls. Total RNA from each sample was prepared for the microarray analysis according to the manufacturer’s protocol (Arraystar, USA). Briefly, total RNA was digested with RNase R (Epicentre, Inc., USA) to enrich circular RNAs. Enriched circular RNAs were amplified and transcribed into fluorescent complimentary RNA utilizing a random priming method (Arraystar Super RNA Labeling Kit; Arraystar, USA) and then hybridized onto the Arraystar Human circRNA Array V2 (8x15K, Arraystar, USA). Slides were washed and the arrays were afterwards scanned by the Agilent Scanner G2505C.

Acquired array images were analyzed using Agilent Feature Extraction software (version 11.0.1.1). Quantile normalization and subsequent data processing were performed using the R software limma package. Differentially expressed circRNAs with statistical significance between two groups were identified through volcano plot filtering. Fold change filtering was used to identify differentially expressed circRNAs between two samples. Distinguishable circRNA expression patterns among samples were identified through hierarchical clustering.

### Real-time Quantitative PCR Validation of circRNA Expression

cDNA synthesis was performed on total RNA samples using SuperScript VILO Master Mix (ThermoFisher, USA). Expression levels of selected circRNAs were measured by real-time quantitative PCR (qPCR) using Sybr Select Master Mix (ThermoFisher, USA) on the Rotor Gene Q 5plex HRM platform (Qiagen, Germany) in duplicate for each sample. Primers for qPCR are shown in Table 2. Primers for RPL13A were synthesized by Qiagen (Germany) and all other were synthesized by IDT (USA). RPS17 and RPL13A were used as reference genes. The data were analyzed using the comparative cycle threshold method (2^ΔΔCt^).

**Table 2:** List of primers for qPCR validation of microarray results

Target RNA	Primer sequence (5′–3′)
hsa_circ_0000567	F: AAACACAGCTCGACAGTACGC
	R: TCCTTTGGTGACACAGTTGC
hsa_circ_0001173	F: TGCAAGGTGAAGTTCAGAGG
	R: TCTGCTGGCAATTCAAACAC
hsa_circ_0005218	F: TACGCAACATTCAGGACACC
	R: GCCATGGAAACCATTCTCTC
hsa_circ_0005896	F: TCAAGATTTTAAGGTCAAGATAGCA
	R: CAATCTATTCAAACATTAGCTTACCA
hsa_circ_0023919	F: ATTTGCAGCAGCCAACTTTT
	R: CCTGCTTGCAGCTGTAGAATC
hsa_circ_0035796	F: CAGGGTGTTTTGGTTTAGGC
	R: GCCTGTTCTTCCATTTCAGC
hsa_circ_0043138	F: ATGATCAGCAGCATGATTCC
	R: ATCAGTCGTTTGCCCATAGC
hsa_circ_0063411	F: CCGTGCAGCCACTAAATTCT
	R: TCCTCCATCCTCCTCCTCTT
hsa_circ_0073647	F: AACACCACACAGAGGCACAG
	R: CCCCAGCAAAGTGTAGCAGT
hsa_circ_0088036	F: TACGTCCGGGTACCAACTAC
	R: CTCCATCTCAAGCAGGTTTC
RPS17	F: CCATTATCCCCAGCAAAAAG
	R: GAGACCTCAGGAACATAATTG
RPL13A	QuantiTect: Hs_RPL13A_1_SG (Cat. No. QT00089915)

### Statistical Analysis

All experimental data were analyzed using SPSS software 24.0 (SPSS, USA). Differences in expression levels between patients and healthy controls were assessed using *t* test or Mann-Whitney *U* test, as appropriate. The correlations between circRNA expression levels and clinical data were determined by Spearman’s rank correlation. ROC curve analysis was performed to assess the diagnostic potential of statistically differentially expressed circRNAs. *p* value < 0.05 was considered to be statistically significant.

## Results

### Microarray Expression Profile

**Fig. 1:**
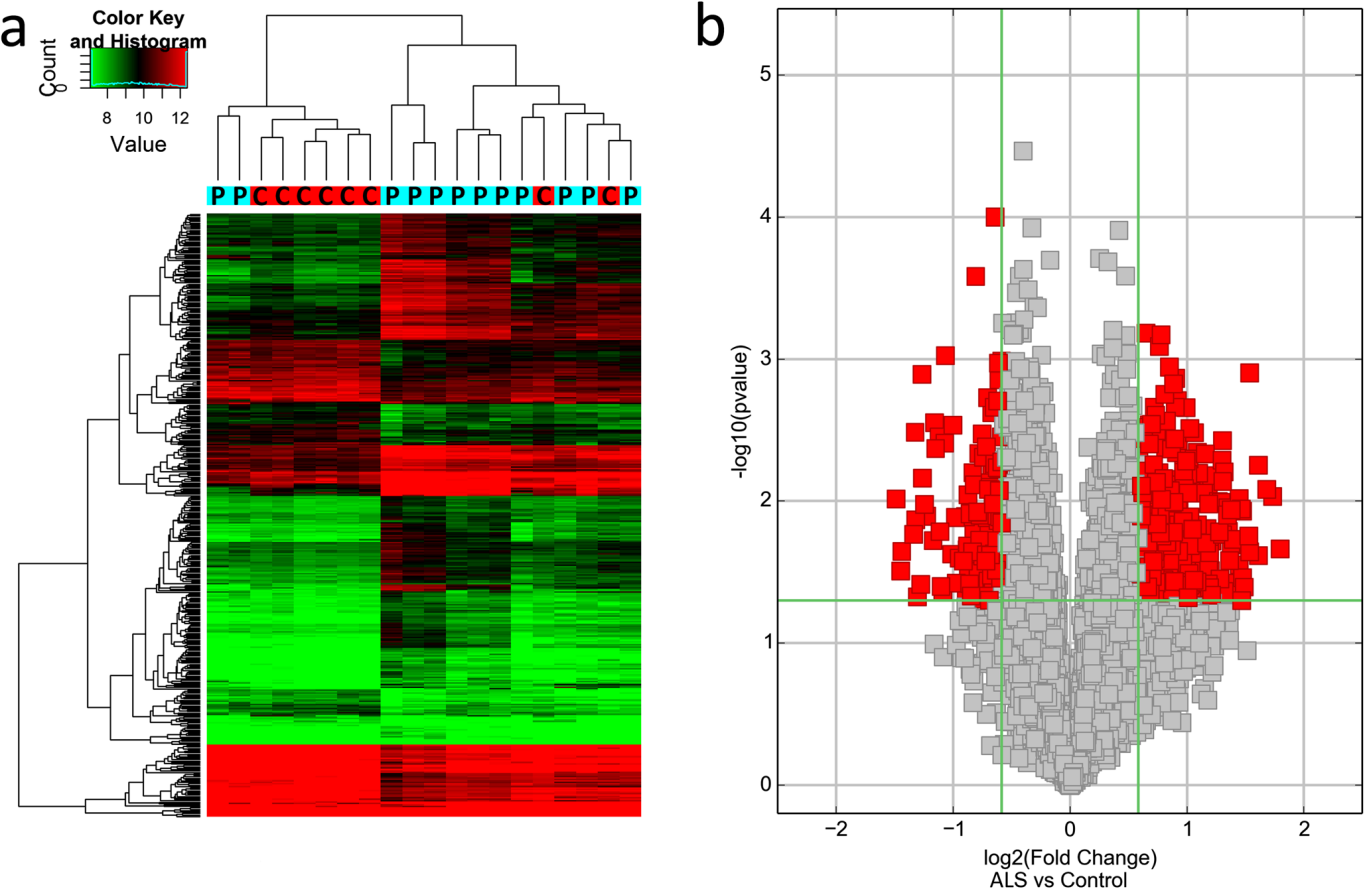
Summary of microarray expression profile. **a** Hierarchical clustering and heatmap visualization of circRNA expression levels. P, ALS patient; C, healthy control. **b** Volcano plot representation of differentially expressed circRNAs (red points; *p* value < 0.05 and fold change > 1.5). Two hundred seventy-four of them were upregulated and 151 were downregulated when comparing ALS patients and healthy controls

Microarray expression profile of circRNAs in ALS was performed on blood samples from 12 ALS patients and 8 age- and sex-matched healthy controls using Arraystar Human circRNA Array Analysis. Hierarchical clustering and subsequent heatmap visualization of circRNA expression levels in samples showed distinguishable expression patterns among healthy controls and ALS patients (Fig. 1a). Moreover, we analyzed differences in expression levels using volcano plot (Fig. 1b) and identified 425 differentially expressed circRNAs when comparing ALS patients and healthy controls (circRNAs with fold change > 1.5 and *p* value < 0.05). Of them, 274 were upregulated and 151 were downregulated in ALS patients.

Following the initial microarray analysis, we selected 10 circRNAs for qPCR validation of their expression. Selection criteria included, but were not limited to, *p* value (< 0.04), fold change (> 1.8), genomic location (exonic), and function of the hosting gene. We evaluated the function of the hosting gene and its potential involvement in ALS through literature and database search (ALSoD [20]: http://alsod.iop.kcl.ac.uk/; Ensembl [21]: release 94). Summary of these criteria for selected circRNAs is shown in Table 3.

**Table 3:** Selected circRNAs for qPCR validation, their microarray information, and reason for validation

circRNA	*p* value (Benjamini-Hochberg FDR)	Fold change	Regulation	Genomic location	Reason for validation
hsa_circ_0000567	0.007	4.00	Down	*SETD3*	SETD3 is histone methyltransferase that regulates muscle differentiation in mouse [22]
hsa_circ_0001173	0.03	1.83	Down	*VAPB*	*VAPB* is ALS-associated gene [23]
hsa_circ_0005218	0.004	2.63	Down	*FAM120A*	FAM120A interacts with HNRNPA1 (associated with ALS) [24]
hsa_circ_0005896	0.04	2.82	Up	*SMN1*	SMN1 is involved in mRNA processing and neurogenesis [25]
hsa_circ_0023919	0.005	3.03	Down	*PICALM*	PICALM is involved in the clathrin-mediated endocytosis at the neuromuscular junctions [26]
hsa_circ_0035796	0.008	5.36	Down	*HERC1*	HERC1 has an extensive role in the neurotransmission at the neuromuscular junctions [27]
hsa_circ_0043138	0.008	5.34	Down	*TAF15*	*TAF15* is ALS-associated gene [28]
hsa_circ_0063411	0.002	3.32	Up	*TNRC6B*	TNRC6B guides Ago-mediated gene silencing [29]
hsa_circ_0073647	0.002	9.64	Up	*SEMA6A*	SEMA6A is involved in axon guidance [30]
hsa_circ_0088036	0.004	4.12	Up	*SUSD1*	SUSD1 is potentially associated with ALS [31]

### qPCR Validation of circRNA Expression

**Fig. 2:**
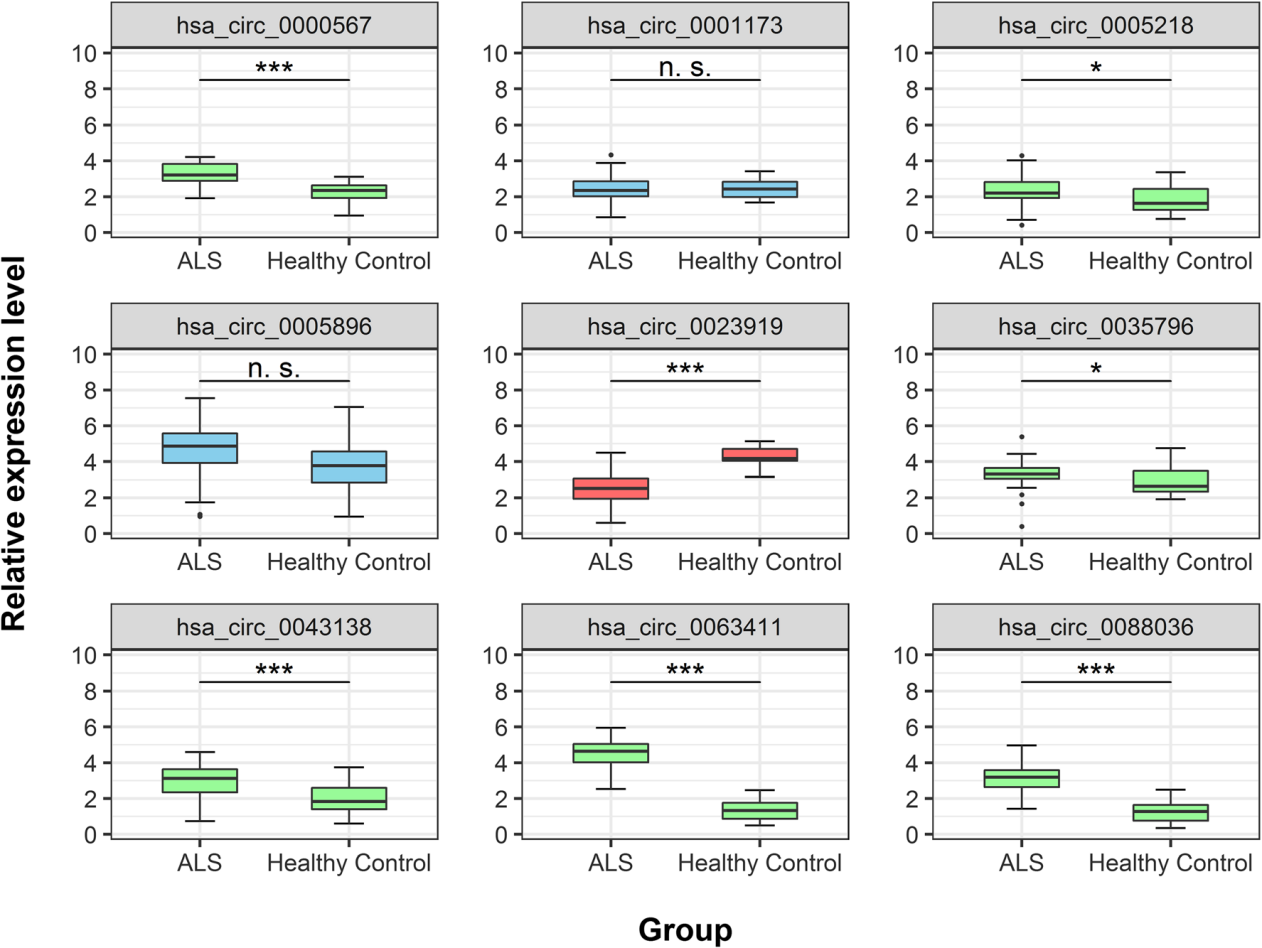
Expression of selected circRNAs in ALS patients and healthy controls. Relative expression levels of each circRNA in ALS patients (*n* = 60) and healthy controls (*n* = 15) are represented with box plot. Upregulated circRNAs are shown in green, downregulated circRNAs in red, and circRNAs with non-significant differences in expression in blue; significant difference in expression levels is denoted as * (*p* < 0.05) or *** (*p* < 0.001); n. s., non-significant

Microarray expression results were validated with qPCR on blood samples from 60 ALS patients and 15 age- and sex-matched healthy controls. Of 10 selected circRNAs, we got 6 significantly upregulated circRNAs (hsa_circ_0000567, hsa_circ_0005218, hsa_circ_0035796, hsa_circ_0043138, hsa_circ_0063411, and hsa_circ_0088036) and 1 significantly downregulated circRNA (hsa_circ_0023919) in ALS patients. hsa_circ_0005896 and hsa_circ_0001173 showed no significant difference in expression between ALS patients and healthy controls and expression levels of hsa_circ_0073647 were not detectable in either ALS patients or healthy controls (Fig. 2). Among significantly dysregulated circRNAs, hsa_circ_0063411 and hsa_circ_0088036 were upregulated in both microarray and qPCR analyses, while hsa_circ_0023919 was downregulated in both experiments. Four circRNAs (hsa_circ_0000567, hsa_circ_0005218, hsa_circ_0035796, and hsa_circ_0043138) showed discrepancy between the results from the two experiments as they were downregulated in microarray analysis and upregulated in qPCR results.

### Associations Between Clinical Variables and circRNA Expression

The Spearman rank correlation test was performed to assess potential associations between circRNA expression and clinical variables. As shown in Table 4, the expression levels of hsa_circ_0000567 and hsa_circ_0088036 were negatively associated with age, both at the time of blood collection and at the time of disease onset. There was no association between the expression of either of these two circRNAs and the age at the time of blood collection in healthy controls (hsa_circ_0000567: Spearman’s rho = − 0.020, *p* = 0.944; hsa_circ_0088036: Spearman’s rho = 0.263, *p* = 0.725). Another negative association was found between the expression of hsa_circ_0023919 and the age, however, only at the time of blood collection. Similarly, there was no association between hsa_circ_0023919 expression and the age at the time of blood collection in healthy controls (Spearman’s rho = − 0.059, *p* = 0.834). The expression levels of hsa_circ_0063411 and hsa_circ_0005218 were positively associated with the level of functional impairment in patients and the expression of hsa_circ_0063411 was negatively associated also with the disease duration and survival time. Also, the expression levels of several circRNA were positively correlated with each other.

**Table 4:** Correlations between circRNA expression levels in ALS patients and association with clinical variables. Spearman rank correlation test revealed moderate negative association between hsa_circ_0000567 and age (both at the time of blood collection and at the time of disease onset). Another moderate negative association was found between hsa_circ_0063411 and disease duration. Expression of several circRNAs is correlated with the expression of other circRNAs. Significant correlations and associations are represented with * (*p* < 0.05) or ** (*p* < 0.01). For the details on clinical characteristics, see Table 1

	Sex	Age at the time of blood collection	ALS onset	Age at onset	Level of functional impairment	Rate of progression	Disease duration	Survival time	hsa_circ_0000567	hsa_circ_001173	hsa_circ_0005218	hsa_circ_0005896	hsa_circ_0023919	hsa_circ_0035796	hsa_circ_0043138	hsa_circ_0063411	hsa_circ_0088036
Sex	–	0.176	0.289*	0.146	0.077	− 0.165	− 0.166	0.134	− 0.019	− 0.152	− 0.085	− 0.011	0.042	− 0.158	0.010	− 0.170	0.077
Age at the time of blood collection		–	0.187	0.991**	− 0.177	− 0.241	− 0.037	0.167	− 0.463**	− 0.203	− 0.084	− 0.131	− 0.283*	− 0.246	− 0.012	− 0.165	− 0.374**
ALS onset			–	0.202	0.288*	− 0.225	− 0.294*	− 0.164	− 0.122	0.033	0.162	− 0.058	− 0.038	− 0.023	− 0.239	− 0.095	− 0.065
Age at onset				–	− 0.148	− 0.307	− 0.113	0.108	− 0.420**	− 0.159	− 0.070	− 0.139	− 0.252	− 0.181	0.047	− 0.118	− 0.346**
Level of functional impairment					–	− 0.177	− 0.418**	− 0.450*	0.125	0.168	0.313*	0.168	0.178	0.202	− 0.022	0.398**	0.116
Rate of progression						–	0.593**	0.643**	0.190	0.247	0.145	0.269	0.081	− 0.173	0.280	− 0.009	0.198
Disease duration							–	0.818*	− 0.075	− 0.087	− 0.119	− 0.056	− 0.126	− 0.240	0.182	− 0.339*	− 0.047
Survival time								–	− 0.170	− 0.185	− 0.225	− 0.099	− 0.101	− 0.322	0.034	− 0.571**	− 0.058
hsa_circ_0000567									–	0.716**	0.438**	0.232	0.625**	0.525**	0.209	0.587**	0.767**
hsa_circ_0001173										–	0.653**	0.383**	0.592**	0.575**	0.136	0.621**	0.614**
hsa_circ_0005218											–	0.418**	0.422**	0.535**	0.075	0.495**	0.430**
hsa_circ_0005896												–	0.509**	0.293*	− 0.048	0.242	0.343*
hsa_circ_0023919													–	0.409**	− 0.107	0.543**	0.593**
hsa_circ_0035796														–	− 0.064	0.529**	0.520**
hsa_circ_0043138															–	0.002	0.222
hsa_circ_0063411																–	0.579**
hsa_circ_0088036																	–

### Determination of circRNA Diagnostic Potential by ROC Curve Analysis

**Fig. 3:**
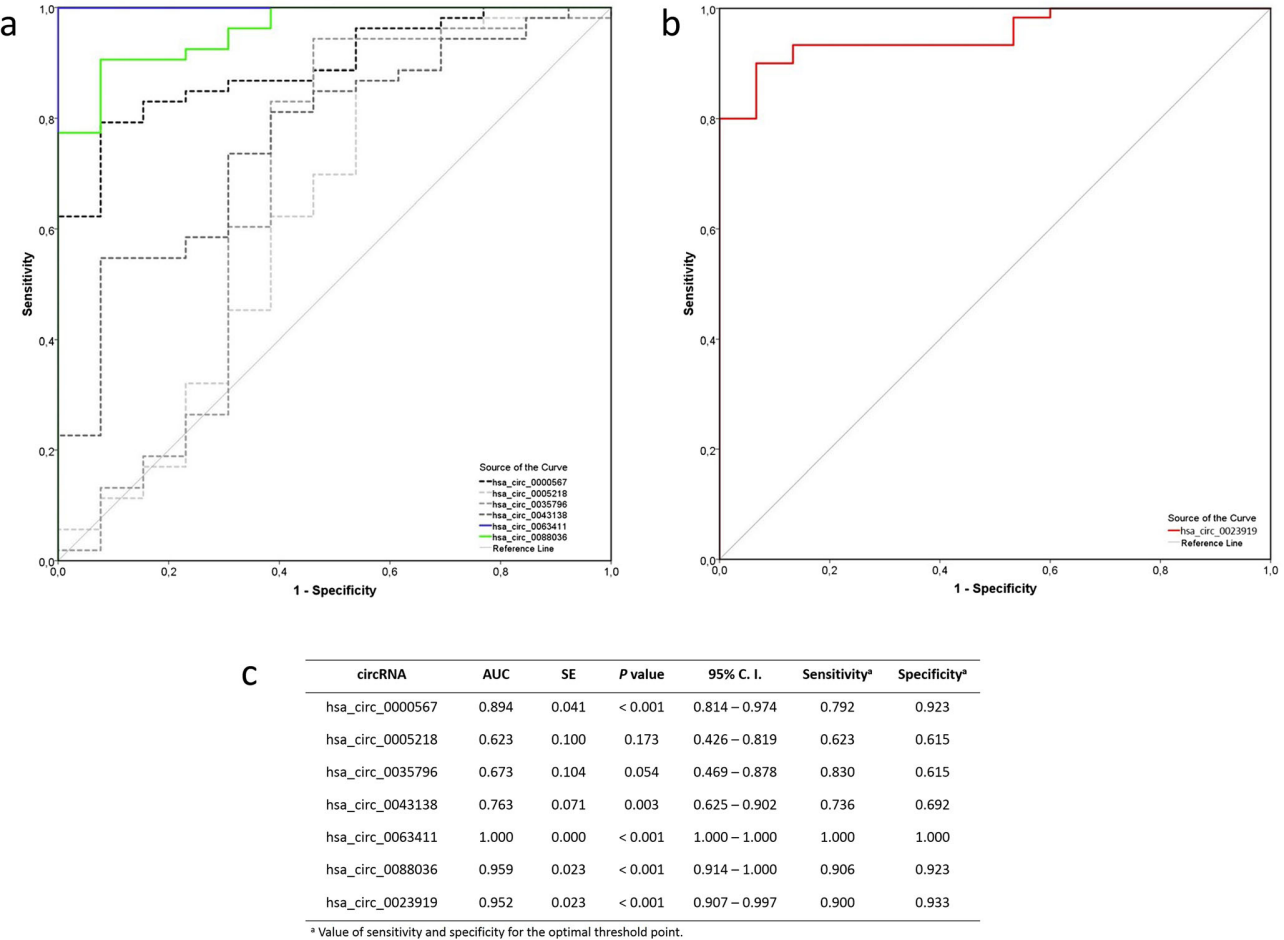
ROC curves for the circRNAs with statistically significant difference in expression. Full colored lines represent circRNAs with AUC > 0.950, dashed gray lines represent other circRNAs. **a** ROC curves for upregulated circRNAs. **b** ROC curve for downregulated circRNA. **c** Details of shown ROC curves. AUC, area under the curve, SE, standard error for AUC, 95% C. I., 95% confidence interval for AUC. Optimal threshold point was determined as the point on the curve with minimal distance to the ideal point (sensitivity = 1 and specificity = 1)

We performed receiver operating characteristics (ROC) curve analysis to evaluate the diagnostic potential of selected circRNAs (Fig. 3). Of 7 circRNAs with statistical difference in expression levels between cases and controls, three (hsa_circ_0023919, red; hsa_circ_0088036, green; hsa_circ_0063411, blue) had area under the curve (AUC) over 0.950 (Fig. 3a, b). These circRNAs also had outstanding specificity and sensitivity at the optimal threshold point—over 90% (Fig. 3c). AUC values for other circRNAs varied between 0.623 and 0.894 (dashed gray lines) (Fig. 3a, c).

## Discussion

circRNAs are widely expressed in several human tissues [32, 33] and have been already implicated in numerous developmental and physiological processes—myogenesis [34], synaptogenesis [35], and cell growth [36]. Inevitably, they are implicated also in pathological processes—tumorigenesis [16, 37], abnormal mRNA splicing [38], and neurodegeneration [18, 19]. However, no ALS-associated circRNA has been identified yet in human samples. Here, we present the first circRNA differential expression analysis in leukocyte samples from patients with amyotrophic lateral sclerosis. Muscle and nervous tissues are the most affected tissues in ALS; however, blood samples are easier to obtain and thus more suitable for diagnostics if reliable biomarkers exist.

Microarray-based circRNA expression profiling on a representative subset of samples revealed 274 upregulated and 151 downregulated circRNAs between ALS patients and healthy controls. Based on the microarray results and our estimated relevant function of a hosting gene in ALS, we selected 10 circRNAs for further validation of expression on a larger set of samples. In total, expression of 7 of 10 selected circRNAs was significant between ALS samples and healthy controls. Four of them (hsa_circ_0000567, hsa_circ_0023919, hsa_circ_0063411, and hsa_circ_0088036) showed the highest significance as well as clinical relevance. In addition, 3 of them (hsa_circ_0023919, hsa_circ_0063411, and hsa_circ_0088036) also showed identical regulation in both microarray and qPCR results.

hsa_circ_0000567 is located in *SETD3* gene, product of which is histone methyltransferase that regulates muscle differentiation in mouse [22]. This circRNA was downregulated in microarray analysis; however, qPCR results showed significant upregulation in ALS cases. According to Morey et al. [39], correlation of microarray and qPCR results is gene specific and can vary considerably. Particularly in the cases where microarray showed downregulation and qPCR validation failed to confirm that, the result discrepancy may be due to variability in array spot intensity or due to increased sample size in qPCR analysis.

hsa_circ_0023919 is located in *PICALM* gene that is involved in clathrin-mediated endocytosis at neuromuscular junctions [26] and single nucleotide polymorphism upstream of the gene has been associated with Alzheimer’s disease [40]. This circRNA was downregulated in microarray analysis and qPCR results also confirmed this. hsa_circ_0023919 sequence contains two binding sites for hsa-miR-9 (imperfect binding site between 61 and 67 bp and 7mer-m8 binding site between 142 and 148 bp) [41]. Upregulation of miR-9 was confirmed in both mouse model of ALS [42] and in human blood samples of ALS patients [43]. By all means, further functional studies are necessary to investigate the potential association between hsa_circ_0023919 and miR-9 in ALS.

hsa_circ_0063411’s host gene, *TNRC6B*, guides Ago-mediated gene silencing [29]. This circRNA was upregulated in both microarray and qPCR analyses. There is no evidence yet on the role of this circRNA in any biological or pathological process. However, it contains one binding site for hsa-miR-647 (7mer-m8 site between 680 and 686 bp) [41]. Some connection between hsa-miR-647 and ALS was already detected in spinal cord samples from ALS patients where miRNA-647 was found downregulated [44]. In order to elucidate the potential associations between circ_0063411 and hsa-miR-647 expression in ALS patients and their common roles in ALS disease initiation and progression, further studies are necessary.

hsa_circ_0088036 is located in *SUSD1* gene that is potentially associated with ALS [31]. hsa_circ_0088036 was, like hsa_circ_0063411, upregulated in microarray and qPCR experiments. Previous study showed that hsa_circ_0088036 (also known as hsa_circRNA_104871) was significantly upregulated in PBMCs from patients with rheumatoid arthritis and may serve as a potential biomarker for its diagnosis [45]. Since this circRNA was significantly upregulated also in this study and diseases have no common cause, we could speculate that hsa_circ_0063411 might somehow be involved in the immune response. Nevertheless, further studies are necessary to confirm that.

All of selected circRNAs (Table 3) have one or more predicted binding sites for several RBPs. Two of them, AGO2 and EIF4A3, can bind to all of the selected circRNAs with the exception of hsa_circ_0035796 that has binding sites only for EIF4A3. Despite potential role of circRNA to serve as scaffolds for protein complexes [14], the variability in the number of binding sites and presence in circRNAs of various origins and functions indicate other potential explanations. As Chen et al. [46] showed, RBPs are involved in discriminating between endogenous and exogenous circRNAs and abolishing immune response to endogenous circRNAs. Origin discrimination is based on intronic sequences that are involved in splicing and circularization and associated with splicing complexes, part of which is also EIF4A3 [46].

Another RNA-binding protein, fused in sarcoma (FUS), has been recognized as an important modulator of circRNA expression [47]. Errichelli et al. observed an overall downregulation of circRNA expression in FUS^−/−^ mice and expression was dysregulated also in FUS^R521C^ and FUS^P525L^ human-induced pluripotent stem cell-derived motor neurons. Since cognate linear transcripts showed no significant alteration in expression levels, circRNA deregulation can be attributed to altered splicing dynamics due to mutated or absent FUS. Whether this is the reason for altered circRNA expression also in human tissues, it remains to be determined. These findings could have considerable implications for further research on circRNAs in ALS as mutations in FUS have been found in 5% of ALS cases [2].

With the Spearman rank correlation test, we evaluated the potential associations between circRNA expression and clinical data or correlations between expression levels of each circRNA. We found that three circRNAs (hsa_circ_0000567, hsa_circ_0023919, and hsa_circ_0088036) were negatively associated with the age of ALS patient at the time of blood collection and two of them (hsa_circ_0000567 and hsa_circ_0088036) were also negatively associated with the age at the disease onset. None of these circRNAs were associated with the age at the time of blood collection in healthy controls, suggesting that this association is disease specific. hsa_circ_0005218 was positively correlated with the level of functional impairment in ALS patients and hsa_circ_0063411 was negatively correlated with disease duration and survival time. Verification of clinically relevant associations is needed to exclude potential influence of other clinical conditions. Moreover, expression of several circRNAs was positively correlated with each other, indicating potential involvement of these circRNAs in similar biological processes and/or co-regulation. Extensive functional studies are of course needed to evaluate these indications.

Through ROC curve analysis, we identified hsa_circ_0023919, hsa_circ_0088036, and hsa_circ_0063411 as potential blood-based biomarkers for ALS. All of them had AUC values above 0.95. Moreover, at the optimal threshold point, each of them had both sensitivity and specificity above 90%. Among already discovered fluid-based biomarkers for ALS are the most promising two protein biomarkers, NFL and pNFH. However, they do not reach such sensitivity and specificity in serum or plasma samples [5] as have reached circRNAs in this study. Therefore, we could speculate that circRNAs hsa_circ_0023919, hsa_circ_0088036, and hsa_circ_0063411 could possess great clinical relevance in ALS. However, studies with increased sample size and more diverse set of controls are needed to justify this. Furthermore, comparison with other neurodegenerative diseases is necessary in order to investigate ALS disease specificity.

In conclusion, to our knowledge, this is the first study of circRNA expression profile in human samples of ALS. It provides a broad framework for further functional studies on the role of circRNAs in ALS. This might help to improve our understanding about the molecular mechanisms in ALS. This work also revealed promising diagnostic potential of circRNAs. We think therefore that circRNAs and their association with ALS are definitely worth to be further investigated.

## Abbreviations

ALS: Amyotrophic lateral sclerosis
ALS-FRS-R: ALS functional rating scale revised
AUC: Area under the curve
circRNA: Circular RNA
FALS: Familial ALS
NFL: Neurofilament light chain
PBMCs: Peripheral blood mononuclear cells
pNFH: Phosphorylated neurofilament heavy chain
RBP: RNA-binding protein
ROC: Receiver operating characteristics
SALS: Sporadic ALS
snRNAs: Small nucleolar RNAs
